# Construction of a WMR for Trajectory Tracking Control: Experimental Results

**DOI:** 10.1155/2013/723645

**Published:** 2013-07-11

**Authors:** R. Silva-Ortigoza, C. Márquez-Sánchez, M. Marcelino-Aranda, M. Marciano-Melchor, G. Silva-Ortigoza, R. Bautista-Quintero, E. R. Ramos-Silvestre, J. C. Rivera-Díaz, D. Muñoz-Carrillo

**Affiliations:** ^1^Instituto Politécnico Nacional, CIDETEC, Área de Mecatrónica, Unidad Profesional Adolfo López Mateos, 07700 México, DF, Mexico; ^2^Instituto Politécnico Nacional, UPIICSA, Sección de Estudios de Posgrado e Investigación, 08400 México, DF, Mexico; ^3^Benemérita Universidad Autónoma de Puebla, Facultad de Ciencias Físico Matemáticas, 72001 Puebla, PUE, Mexico; ^4^Instituto Tecnológico de Culiacán, Departamento de Metal-Mecánica, 80220 Culiacán, SIN, Mexico; ^5^Universidad Privada del Valle, Facultad de Informática y Electrónica, Tiquipaya, CBBA, Bolivia; ^6^Centro Nacional de Actualización Docente, Área de Máquinas, 13420 México, DF, Mexico

## Abstract

This paper reports a solution for trajectory tracking control of a differential drive wheeled mobile robot (WMR) based on a hierarchical approach. The general design and construction of the WMR are described. The hierarchical controller proposed has two components: a high-level control and a low-level control. The high-level control law is based on an input-output linearization scheme for the robot kinematic model, which provides the desired angular velocity profiles that the WMR has to track in order to achieve the desired position (x∗, y∗) and orientation (*φ*∗). Then, a low-level control law, based on a proportional integral (PI) approach, is designed to control the velocity of the WMR wheels to ensure those tracking features. Regarding the trajectories, this paper provides the solution or the following cases: (1) time-varying parametric trajectories such as straight lines and parabolas and (2) smooth curves fitted by cubic splines which are generated by the desired data points {(x1∗, y1∗),..., (xn∗, yn∗)}. A straightforward algorithm is developed for constructing the cubic splines. Finally, this paper includes an experimental validation of the proposed technique by employing a DS1104 dSPACE electronic board along with MATLAB/Simulink software.

## 1. Introduction

In the last decades, the control of wheeled mobile robots (WMRs) has been an interesting topic for research [1]. The differential robot configuration studied in this paper has nonholonomic constraints [2]. In order to improve the autonomy of the mobile robots, the literature in this field has generally focused on solving the following problems: (1) mobile robot positioning, (2) stabilization, (3) trajectory tracking control, (4) planning the trajectories, and (5) obstacle avoidance.

In the robot stabilization problem, according to [3], it is known that a nonholonomic system cannot be asymptotically stabilized at an equilibrium point using a differentiable control law, despite the system's being completely controllable. Accordingly, the stabilization of nonholonomic systems can only be achieved by nondifferentiable control laws [4] or time-dependent ones [5–9].

On the other hand, the trajectory tracking task in nonholonomic systems can be performed through differentiable control laws. In [10], a hierarchical control scheme based on two levels (high-level and low-level) is presented for the trajectory tracking control of a car-trailer system. The high-level control is based on a time-varying linear quadratic regulator, which provides the desired angular velocity profiles that the system has to track in order to achieve the desired trajectory. Then, a low-level control is designed for controlling the traction and the steering motors by using a proportional integral derivative (PID) control. Experimental results from the application of this tracking control scheme are presented. Similarly, in [11], the control of a differential WMR is proposed via a variable structure control scheme, based on external and internal control strategies. The external control is associated to the kinematic model of the mobile robot, which is responsible for generating the desired angular velocity profiles for the motors, via an input-output linearization scheme. Meanwhile, the internal control is meant to track the velocities imposed by the external control via a PID control. In [12], via the differential flatness property of the WMR kinematic model, the dynamic controller design for trajectory tracking tasks was presented. The basis of flatness-based control can be found in the work by Fliess et al. [13]. Likewise, in [14], a sliding mode control in combination with differential flatness was presented for different types of WMRs for tracking problems. These controllers were implemented through computer simulations. In [15], a control algorithm to achieve trajectory tracking of a WMR with nonholonomic constraints was proposed by using the computed torque method and the theory of sliding mode control. The proposed algorithm was implemented on a vision-based mobile robot system. In [16], an input-output linearization design was presented in which the actuators are controlled by a sensorless scheme. More recently, in [17], a formal justification was presented for use of velocity and electric current inner loops, driven by proportional controllers, when a trajectory tracking task is to be solved. In this work, the dynamics of the brushed DC motors used as actuators is taken into account to perform the stability analysis.

Different approaches to the trajectory generation problem have been widely studied (see, e.g., [18]) where the “path planning” problem is related to generating a sequence of points through which the robot must pass (either an end-effector of an industrial robot or a mobile trajectory); see [19]. Another approach is known as “trajectory planning and optimization,” which includes a structure that integrates the dynamics with the path planning. Multiobjective optimization can be addressed, such as the cost efficient use of energy, the task time, and other similar constraints; see [20]. Although the trajectory generation can be solved regardless of dynamic consideration, real constraint issues make this approach impractical for many applications. An ideal trajectory implementation with smooth transitions (human-like) as well as being robust against external disturbances must be designed under multiobjective contradictory goals. A well known implementation for this problem is based on splines (see [21–23]) which can be designed in such a way that both the velocity and acceleration transitions follow the dynamic requirements. For instance, in [24, 25], an algorithm for path planning for mobile robots used in football competitions was presented. In [26], a technique was shown for path planning based on splines, which was used to simulate differential mobile robots.

The contribution of this research is to present an integration of both theoretical and practical knowledge in order to close the gap between the two. The relevant and modern open-architecture testbed described in this paper deals with a synergetic combination of an effective control approach and a state-of-the-art technology for rapid prototyping that combines a hard real-time control implementation with a software (MATLAB/Simulink) widely used for these applications. In this context, the present paper has three aims: (1) to describe the construction of a differential drive WMR, (2) to show the implementation of a real-time hierarchical control strategy in order to carry out a trajectory tracking control task, and (3) to present a methodology for generating the WMR trajectories (based on cubic splines) which are constructed from the desired data points {(*x*_1_*, *y*_1_*),…, (*x*_n_*, *y*_n_*)}. Furthermore, time-varying parametric trajectories such as straight lines and parabolic curves, are also implemented.

To this end, the present paper is organized as follows.  Section 2 presents the general description of the WMR construction.  Section 3 describes the hierarchical controller law for the kinematic model for the trajectory tracking task.  Section 4 gives an algorithm for generating smooth curves based on specific points given in the *X*-*Y* plane via cubic splines.  Section 5 shows the real-time control implementation for the WMR. Finally, some conclusions and prospects for future research are presented in Section 6.

## 2. Construction of the WMR

**Figure 1:**
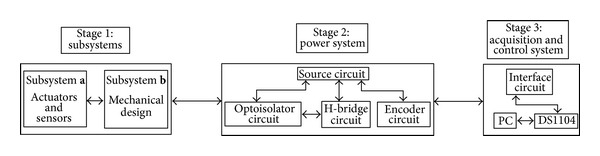
General block diagram of the WMR prototype.

 A mobile robot is, in general, composed of two mechanical subsystems: (1)* actuators and sensors* and (2)* mechanical design*. The control of these subsystems requires two electronic stages: (1) the power stage and (2) the acquisition and control stage. The interaction of these stages is shown in the block diagram presented in Figure 1. The electronic power stage (stage 2) allows interaction between the electronic control interface (stage 3) and the two dynamic subsystems (stage 1). This interaction includes the communication system, the power supply, and the conditioning circuits (in order to interconnect the electronic board DS1104). The strategy used in the PC (using MATLAB/Simulink) keeps the whole system under control, taking into consideration the physical restrictions. Likewise, the control stage comprises the control strategies used to integrate the functioning of each subsystem (based on mathematic models of the plant). 

### 2.1. Stage 1: Subsystems

 This part describes the WMR dynamic subsystems **a** and **b**, which include actuators, sensors, and the mechanical structure. Subsystem **a** (motors and sensors) allows propulsion of the WMR in a specified workspace and discrete position sensing. The subsystem **b** describes the mechanical design.

#### 2.1.1. Subsystem a**:**  Actuators and Sensors

The prototype described in this work uses DC motors as actuators. In order to estimate the mechanical capacity of these actuators, it is proposed to use the maximum velocity, acceleration, and weight of the WMR system as

(1) $$\begin{array}{c} \upsilon =1\;\text{m}/\text{s},\;\;a=1\;\text{m}/{\text{s}}^{2},\;\;m=46.74\;\text{kg}. \end{array}$$

Based on the mass *m* and maximum acceleration *a*, it is possible to calculate the required force *F* for a given displacement as

(2) $$\begin{array}{c} F=ma=\left(46.74\;\text{kg}\right)\left(1\;\text{m}/{\text{s}}^{2}\right)=46.74\;\text{N}. \end{array}$$

Since two motors are used, each of them has an associated force, *F*_rm_ for the right wheel motor and *F*_lm_ for the left wheel motor. One motor provides half of the total force required, that is,

(3) $$\begin{array}{c} F_{\text{rm}}=F_{\text{lm}}=\frac{F}{2}=\frac{46.74\;\text{N}}{2}=23.37\;\text{N}. \end{array}$$

Since the wheel diameter is 0.15 m (*r* = 0.075 m), the required torque for each motor is given by

(4) $$\begin{array}{c} \tau_{\text{rm}}=F_{\text{rm}}r=\left(23.37\;\text{N}\right)\left(0.075\;\text{m}\right)=1.75\;\text{Nm},\\ \tau_{\text{lm}}=F_{\text{lm}}r=\left(23.37\;\text{N}\right)\left(0.075\;\text{m}\right)=1.75\;\text{Nm}. \end{array}$$

Since *υ* = *ω*_w_ *r*, the angular velocity is related to each wheel and it is determined by

(5) $$\begin{array}{c} \omega_{\text{w}}=\frac{\upsilon }{r}=\frac{1\;\text{m}/\text{s}}{0.075\;\text{m}}\approx 13.33\;\text{rad}/\text{s}\approx 127.35\;\text{rpm}. \end{array}$$

Hence, the power required, *P*_w_, is calculated as follows:

(6) $$\begin{array}{c} P_{\text{w}}=\tau_{\text{rm}}\omega_{\text{w}}=\left(1.75\;\text{Nm}\right)\left(13.33\;\text{rad}/\text{s}\right)\approx 23.37\;\text{W}. \end{array}$$

The energy must be transmitted to each wheel of the WMR; however, the power required is higher than the calculated power since there is a loss due to the gearbox coupling. The gearbox efficiency is around 70%, so that the estimation of the power required (*P*_*d*_) is given by

(7) $$\begin{array}{c} P_{d}=\frac{P_{\text{w}}}{0.7}=\frac{23.37\;\text{W}}{0.7}=33.39\;\text{W}. \end{array}$$

Based on the data sheet of the motor [27] and the golden rule for selecting an appropriate motor, it is recommended to have between 1.5 and 2 times the required power *P*_*d*_, and thus

(8) $$\begin{array}{c} 50.09\;\text{W}<P_{d}<66.78\;\text{W}, \end{array}$$

so that the motors selected are *GNM 3150* (24 V, 55 W) and the gearbox chosen for each motor is *G 2.6*. The second golden rule given by the manufacturer shows the importance of maintaining the desired velocity *ω*_*d*_ between 65% and 90% of the no-load motor velocity (i.e., 3526 rpm), and thus

(9) $$\begin{array}{c} 2291.9\;\text{rpm}<\omega_{d}<3173.4\;\text{rpm}. \end{array}$$

In order to satisfy this condition, the reduction ratio should be at least 20 : 1, since

(10) $$\begin{array}{c} \omega_{m}=20\omega_{\text{w}}=\left(20\right)\left(127.35\;\text{rpm}\right)=2547\;\text{rpm}. \end{array}$$

Finally, these selected motors, including the gearboxes, are the series *GNM 3150* (24 V, 55 W) Engel brand and a gearbox *G 2.6*. The power for the motors is supplied with two YUASA batteries 12 V @ 12 Ah.

Regarding the prototype sensors, it uses two incremental optical encoders. These sensors allow estimating the angular velocity of the wheels, and consequently the robot is able to perform the trajectory tracking task. The manufacturer of the sensors is the Korean *Autonics*, and the model series is E50S8. These sensor are powered by the YUASA battery previously presented. The configuration of these sensors in the prototype can be seen in Figure 9.

#### 2.1.2. Subsystem b**:**  Mechanical Design

**Figure 2:**
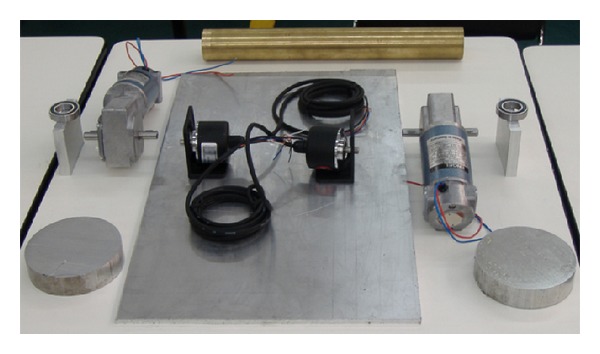
Materials used for the prototype construction.

 Once the components were selected (motors, encoders, and batteries), the software SolidWorks is used to integrate all the mechanical parts of the WMR into a virtual model. The 3D visual feature of this software allows the correct component distribution in the WMR in accordance with the design requirements. Also, the software can be used for specifying the material properties to the model of each part of the WMR. The parts built for this prototype, from the materials previously selected (see Figure 2, and Videos S1, S2 in Supplementary Material available online at http://dx.doi.org/10.1155/2013/723645), are as follows.

**Figure 3:**
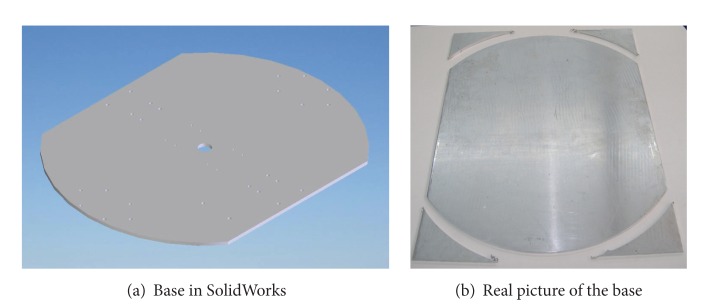
Base design.

**Figure 4:**
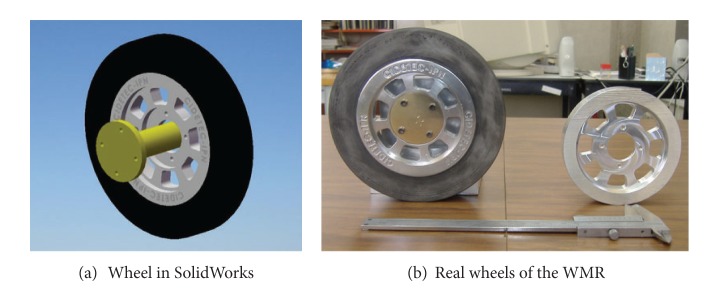
Traction wheels design.

**Figure 5:**
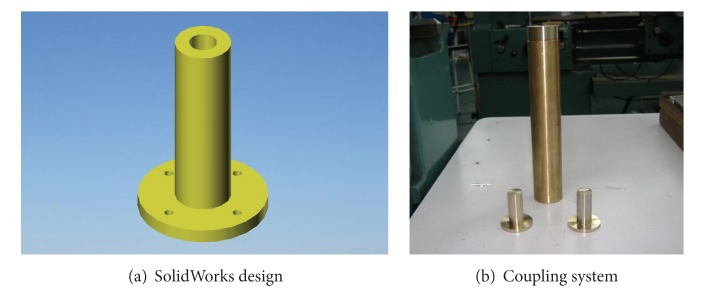
Join coupling system.

**Figure 6:**
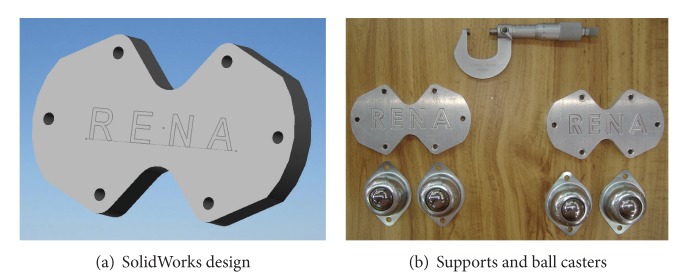
Ball-caster support.

**Figure 7:**
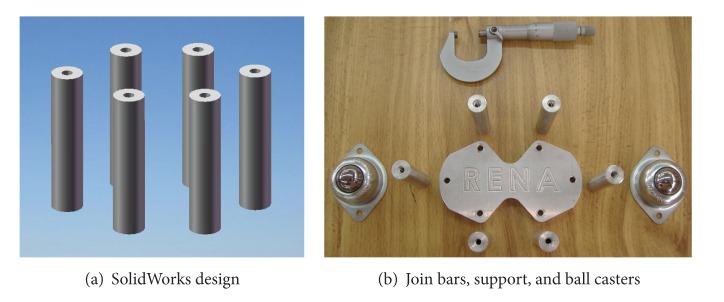
Holding bars design.

**Figure 8:**
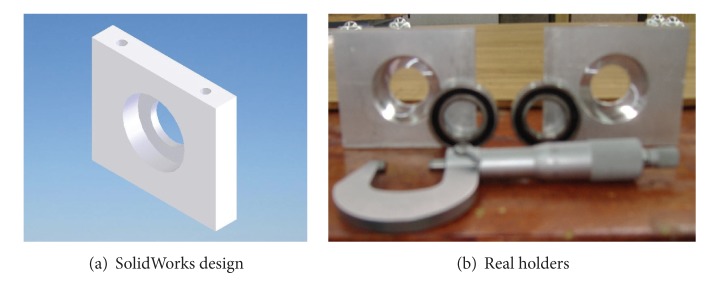
Ball-bearing supports.

 (i) The WMR base is made of aluminum sheet, which is used to support all components (see Figure 3).
 (ii) The wheels used to provide traction to the WMR are constructed with an aluminum rod (see Figure 4).
 (iii) The axle housings are made of yellow brass: they are used for coupling between the shaft of the motor and the wheels (see Figure 5).
 (iv) Supports for the ball casters: they are two rectangular pieces made of aluminum used for assembly the WMR structure (see Figure 6).
 (v) Six aluminum bars join the ball caster supports with the base of the WMR (see Figure 7).
 (vi) Ball-bearings support: the ball bearings allow rotation without translation of the traction wheels due to the shaft motor rotation. Supports have two functions: keeping the ball-bearing embedded into it and joining the base with the motors. It was made of aluminum cut to the appropriate dimensions. A mechanical lathe was used for making the box in the supports in order to place the ball bearing (see Figure 8).

**Figure 9:**
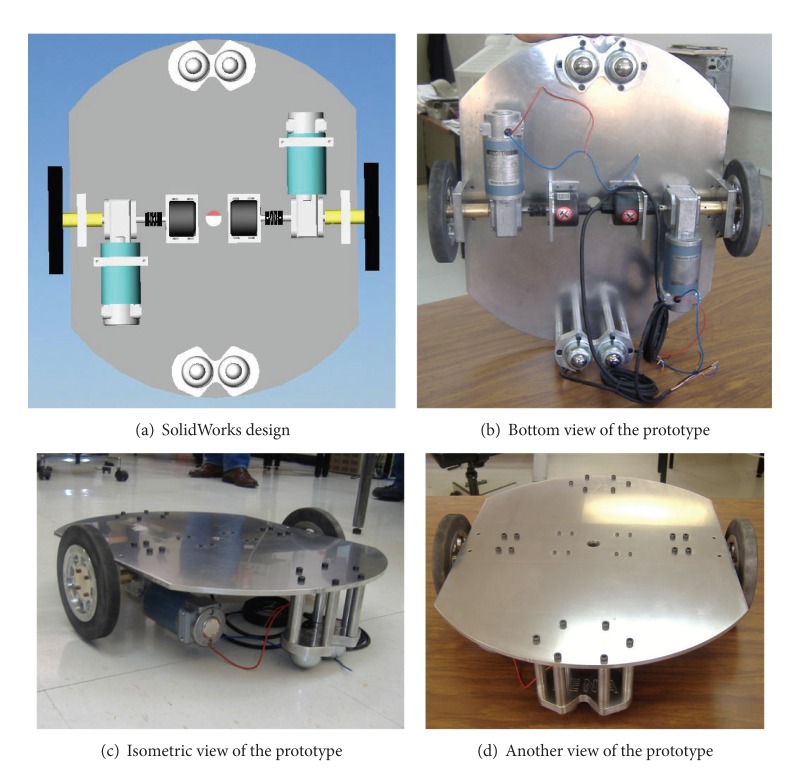
Views of both mechanical design and the real prototype.

To conclude this subsection, Figure 9 shows a bottom view of the final mechanical design made in SolidWorks, as well as pictures of three different views of the prototype.

### 2.2. Stage 2: Power System

 Electronic design is an essential stage for the WMR since its correct functioning depends on it. This section describes the most important features related to the interconnection between the data acquisition board (from the computer) and the conditioning signals (to the WMR).

**Figure 10:**
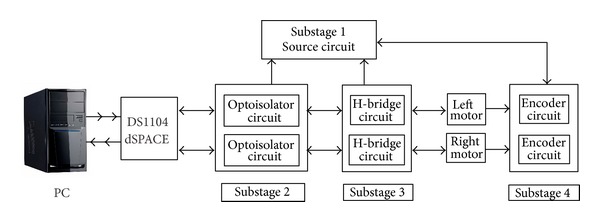
Block diagram of the power stage.

A block diagram of stage 2 is shown in Figure 10. This block makes reference to four substages, numbered from 1 to 4: (*source circuit*, *optoisolator circuit*, *power circuit*, and *encoder circuit*). In *substage 1*, the power supply (source circuit) distributes the different voltages to the general electronic system. *Substage 2* allows electrical signal isolation between the electronic board DS1104 and *substage 3*. Also, this part of the system allows a voltage to be applied across the motor in either direction. This circuit is based on the H-bridge LMD18200. *Substage 4* is used to acquire the encoders' signals, which can then be used to estimate the position in the workspace.

### 2.3. Stage 3: Data Acquisition and Control

**Figure 11:**
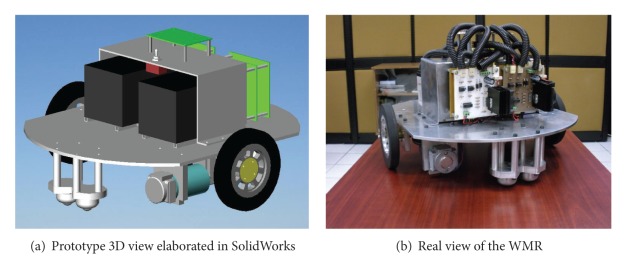
The complete prototype.

 This stage contains the last subsystem presented in the general description of the WMR (see Figure 1). As was mentioned before, the DS1104 board performs the acquisition and control of the WMR. This board was selected due to the integration software between the MATLAB and the board firmware. The high programming level of Simulink (as part of MATLAB) is a practical choice for programming complex control strategies in a graphic environment. This stage also includes the interface circuit (see Figure 1). This circuit establishes the communication between the acquisition board and the WMR. It contains an integrated circuit (74HC541) as a buffer that not only allows the signal bit interchange between the WMR and the DS1104 but also activates two signals DIR and PWM motor (LEDs indicators). Lastly, Figure 11(a) shows the final WMR mechanical design and Figure 11(b) is the real WMR with the instrumentation included (see Video S3 placed online as Supplementary Material).

## 3. Control for the WMR

In this section, two separate controllers are proposed, the first for the kinematic model of the WMR and the second for the DC motors (WMR actuators). The integration of both control strategies is based on the hierarchical control as in [10, 16].

### 3.1. Control for the WMR via Input-Output Linearization

 The WMR studied in this work has two back wheels (left and right) which are identical, completely parallel, inflexible, and joined by a shaft. Moreover, it has front ball casters that make sure that the WMR platform moves in a plane. Assuming that the movements of the WMR are over the *X*-*Y* plane, and that there are no slippery condition, the kinematic model is given by [12]

**Figure 12:**
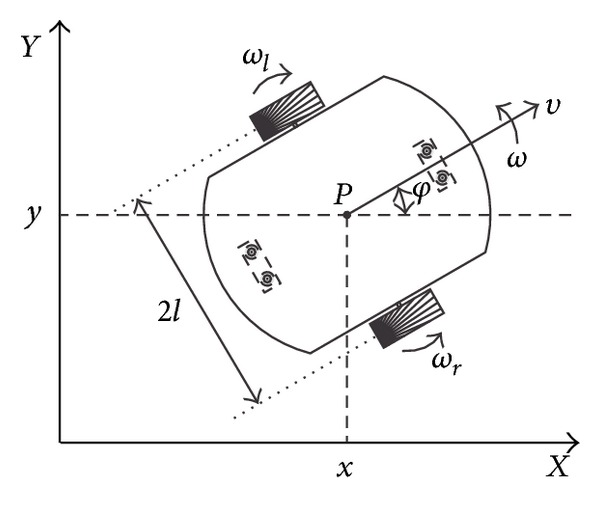
WMR diagram.

(11) x˙=(ωr+ωl)r2cos⁡φ,y˙=(ωr+ωl)r2sinφ,φ˙=(ωr−ωl)r2l,

where (*x*, *y*) is the position measured at the midpoint between the two back wheels, *φ* is the angle between the axis of symmetry of the WMR with respect to the positive *X* axis, and *ω*_*l*_ (*ω*_*r*_) is the angular velocity of the left (right) wheel. Similarly, *r* is the wheel ratio and 2*l* is the gap between them (see Figure 12). In (11), the first derivative with respect to time *t* is denoted by a dot. 

The input control is given by the angular velocity (*ω*_*r*_, *ω*_*l*_) and a strategy based on input-output linearization static feedback. The control can be implemented for any pair of outputs: (*x*, *y*),  (*x*, *φ*), or (*y*, *φ*). Consequently, in each case there will be a remaining dynamic (dynamic zero) so that, in order to ensure a closed loop stable system, it requires an analysis of its stability.

The control design is performed over the output variables (*x*, *φ*) and the analysis of the remaining dynamic in closed loop is studied, particularly for the state variable *y*. First, the kinematic equations that describe the WMR are rewritten in two subsystems. The first is given by

(12) $$\begin{array}{c} \left(\begin{array}{c} \dot{x}\\ \dot{\varphi } \end{array}\right)=A_{1}\left(\begin{array}{c} \omega_{r}\\ \omega_{l} \end{array}\right),\;A_{1}=\left(\begin{array}{cc} \frac{r\cos\varphi }{2} & \frac{r\cos\varphi }{2}\\ \frac{r}{2l} & -\frac{r}{2l} \end{array}\right). \end{array}$$

This model only includes the state variables to be controlled (*x*, *φ*). And, the second is given by

(13) $$\begin{array}{c} \dot{y}=\frac{\left(\omega_{r}+\omega_{l}\right)r}{2}\sin\varphi . \end{array}$$

It has a remaining dynamic associated to the state variable *y*, once the states (*x*, *φ*) have been controlled, so that *x* → x* and *φ* → φ*.

Since det⁡(*A*_1_) = −*r* ^2^cos⁡*φ*/2*l*, a controller can be proposed where the output is (*x*, *φ*) except when *φ* = *kπ*/2, where *k* = ±1,  ±3,  ±5,…, (i.e., *A*_1_ must be invertible). In order to obtain the relation between the controllers (*ω*_*r*_, *ω*_*l*_) and the output variables (*x*, *φ*) from (12), we have

(14) $$\begin{array}{c} \left(\begin{array}{c} \omega_{r}\\ \omega_{l} \end{array}\right)=\left(\begin{array}{cc} \frac{1}{r\cos\varphi } & \frac{l}{r}\\ \frac{1}{r\cos\varphi } & -\frac{l}{r} \end{array}\right)\left(\begin{array}{c} \dot{x}\\ \dot{\varphi } \end{array}\right). \end{array}$$

From these input-output relations, we obtain that the controls (*ω*_*r*_, *ω*_*l*_) that allow the states (*x*, *φ*) to tend asymptotically to the desired trajectory (x*,  φ*) can be defined as follows:

(15) $$\begin{array}{c} \left(\begin{array}{c} \omega_{r}\\ \omega_{l} \end{array}\right)=\left(\begin{array}{cc} \frac{1}{r\cos\varphi } & \frac{l}{r}\\ \frac{1}{r\cos\varphi } & -\frac{l}{r} \end{array}\right)\left(\begin{array}{c} u_{x}\\ u_{\varphi } \end{array}\right), \end{array}$$

where *u*_*x*_ and *u*_*φ*_ are the two auxiliary control variables, written as

(16) ux=x˙∗−αx(x−x∗),uφ=φ˙∗−αφ(φ−φ∗).

Hence, the tracking error dynamics in closed loop is determined by the following linear differential equation system:

(17) e˙x+αxex=0,e˙φ+αφeφ=0,

where *e*_*x*_ = *x* − x* and *e*_*φ*_ = *φ* − φ* denote the tracking errors of the variables *x* and *φ*, respectively, and *α*_*x*_ and *α*_*φ*_ are two positive constants. From (17) it is observed that (*e*_*x*_, *e*_*φ*_) → (0,0) as *t* → *∞*, thus (*x*, *φ*) → (x*, φ*), which is the final control requirement.

Now, regarding the remaining dynamic associated with the state variable *y*, we shall analyze the behavior of this variable when (*x*, *φ*) → (x*,  φ*). For this purpose, in (13), *φ* is replaced by φ* and the controls *ω*_*r*_ and *ω*_*l*_, which are given by (15) and (16) respectively, are substituted, obtaining

(18) $$\begin{array}{c} \dot{y}=\left[{\dot{x}}^{*}-\alpha_{x}\left(x-x^{*}\right)\right]\tan\varphi^{*}. \end{array}$$

Thus, when *t* → *∞*,

(19) $$\begin{array}{c} \dot{y}={\dot{x}}^{*}\tan\varphi^{*}. \end{array}$$

If x* and y* satisfy

(20) $$\begin{array}{c} y^{*}=f\left(x^{*}\right), \end{array}$$

where *f*(x*) is a smooth function such that *f*(0) = 0, then (11) and (20) show that

(21) $$\begin{array}{c} \varphi^{*}=\arctan\left(\frac{{\dot{y}}^{*}}{{\dot{x}}^{*}}\right)=\arctan\left(\frac{df\left(x^{*}\right)}{dx^{*}}\right), \end{array}$$

and (19) can then be simplified as

(22) $$\begin{array}{c} \dot{y}={\dot{x}}^{*}\tan\varphi^{*}={\dot{x}}^{*}\left(\frac{df\left(x^{*}\right)}{dx^{*}}\right)=\frac{df\left(x^{*}\right)}{dt}. \end{array}$$

Now, integrating gives

(23) $$\begin{array}{c} y\left(t\right)=f\left(x^{*}\left(t\right)\right)=y^{*}, \end{array}$$

where the constant of integration has been chosen in such a way that *y*(0) = *f*(0) = 0.

 In conclusion, it has been shown that under the initial condition (*x*, *y*, *φ*) = (0,0, 0), the controllers (15) arrange that the state-space variables (*x*, *y*, *φ*) tend to (x*, y*, φ*), respectively.

### 3.2. PI Control for the DC Motor

 In general, due to the fact that motor manufacturers do not always providing the dynamic parameters of their products, there are different strategies for experimentally obtaining such parameters. This is beyond the scope of this paper; however, there is a reduction from a second-order linear system (motor model: voltage to angular position) to a first-order linear system (motor model: voltage to angular velocity). This can be obtained with the following transfer function: *G*(*s*) = *ϖ*(*s*)/*u*(*s*). For that, Laplace transformation of the motor model is obtained, that is,

(24) $$\begin{array}{c} G\left(s\right)=\frac{\varpi \left(s\right)}{u\left(s\right)}=\frac{nk_{m}}{\left(Js+b\right)\left(Ls+R\right)+n^{2}k_{e}k_{m}}. \end{array}$$

Assuming that inductance may be neglected, *L* ≈ 0, (24) simplifies

(25) $$\begin{array}{c} G\left(s\right)=\frac{\varpi \left(s\right)}{u\left(s\right)}=\frac{K}{\tau s+1}, \end{array}$$

where

(26) $$\begin{array}{c} K=\frac{nk_{m}}{\left(bR+n^{2}k_{e}k_{m}\right)},\;\;\tau =\frac{JR}{\left(bR+n^{2}k_{e}k_{m}\right)}, \end{array}$$

with *u* being the armature motor voltage, *k*_*e*_ the counter-electromotive force constant, *k*_*m*_ the torque constant, *R* the armature constant, *J* the rotor and inertial load, *b* the Coulomb friction coefficient (due to motor and load), and *n* represents the gearbox ratio.

 In order to characterize the dynamic system (i.e., in order to obtain the values of the parameters for the DC motor and load), it is proposed to use a step input function with an amplitude of *A* in order to get coefficients *K* and *τ* such that

(27) $$\begin{array}{c} u\left(s\right)=\frac{A}{s}. \end{array}$$

Substituting (27) into (25) and assuming that the system is a DC motor of which the output has the angular velocity *ϖ*, we obtain the following expression:

(28) $$\begin{array}{c} \varpi \left(s\right)=\frac{K}{\tau s+1}\frac{A}{s}. \end{array}$$

Since the coefficients *K* and *τ* are obtained experimentally using the time representation given by the inverse Laplace transformation of (28) (see (29)), we have

(29) $$\begin{array}{c} \varpi \left(t\right)=KA\left(1-e^{-t/\tau }\right). \end{array}$$

**Figure 13:**
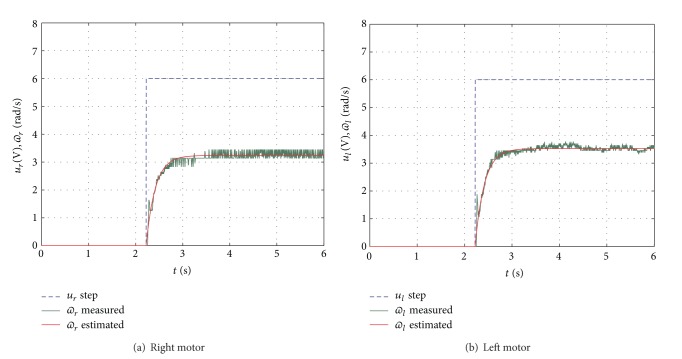
Step input response *u*_*r*_(*t*) = *u*_*l*_(*t*) = 6 V.

Once the characterization of the motor parameters *K* and *τ* is finished, from (25), with *u*(*t*) = 6 V, the experimental results are shown in Figure 13. The characterization of the right motor is

(30) $$\begin{array}{c} K_{r}=0.54,\;\;\tau_{r}=0.10. \end{array}$$

Meanwhile for the left motor, we have

(31) $$\begin{array}{c} K_{l}=0.59,\;\;\tau_{l}=0.10. \end{array}$$

Based on this, the temporal representation of the transfer function for the right and left motors is as follows:

(32) dϖrdt=−10.20ϖr+5.51ur,dϖldt=−10.20ϖl+5.99ul.

Additionally, Figures 13(a) and 13(b) show the step input response of (29) (*ϖ* is labelled as the theoretical value) and the experimentally obtained values of *K* and *τ*. The validation of this characterization is carried out by comparing the theoretical and experimental responses. 

 Since the motors are mechanically attached to the wheels, the angular velocities *ϖ*_*r*_ and *ϖ*_*l*_ must be tracked in order to follow a desired velocity path *ϖ*_r_* and *ϖ*_l_* defined by (15). In order to attain this goal, a PI control is implemented for each motor, so that

(33) $$\begin{array}{c} u\left(t\right)=K_{p}e\left(t\right)+K_{i}\overset{t}{\underset{0}{\int }}e\left(t\right)dt, \end{array}$$

(34) $$\begin{array}{c} e\left(t\right)=\overset{-}{E}\left(t\right)-R\left(t\right)=\varpi^{*}-\varpi , \end{array}$$

where *e*(*t*) is the tracking error, $\overset{-}{E}\left(t\right)$ is the desired value, *R*(*t*) is the signal meant to be controlled, *K*_*p*_ is the proportional gain, and *K*_*i*_ is the integral gain.

DC motors have a nonlinear response due to friction and a dead zone in which the motor requires a certain amount of voltage before it can rotate. However, the dead-zone varies, depending on the mechanical load. In practice, the motor selected for this application requires 1.5 V. For this reason, a friction compensator has to be implemented. A friction model can use a linear gain *f*_*υ*_ (which represents the viscous friction coefficient) and a discontinues shifting phase *f*_*c*_ (which represents the Coulomb friction coefficient) as

(35) Vcomp(ϖ)=[fυabs(ϖ)+fc]sign⁡(ϖ)=fυϖ+fcsign⁡(ϖ),

where *ϖ* is the angular velocity that has to be compensated and *V*_comp_(*ϖ*) is the friction compensator term. In practice, *f*_*υ*_ represents the system viscous friction, and thus

(36) $$\begin{array}{c} f_{\upsilon }=b=n^{2}b_{m}+b_{L}, \end{array}$$

where *b*_*m*_ is the viscous friction coefficient due to the rotor and *b*_*L*_ is the viscous friction coefficient due to the load. In general, these two coefficients values are too small to be estimated accurately; hence, *f*_*υ*_ can be neglected, and thus *f*_*c*_ is taken into account, so that,

(37) $$\begin{array}{c} V_{\text{comp}}\left(\varpi \right)=f_{c}\mathrm{sign}\left(\varpi \right). \end{array}$$

Using (33), the PI controller can be redefined in order to consider the friction compensator *V*_comp_, so that

(38) $$\begin{array}{c} u=K_{p}e\left(t\right)+K_{i}\overset{t}{\underset{0}{\int }}e\left(t\right)dt+V_{\text{comp}}\left(\varpi \right). \end{array}$$

**Figure 14:**
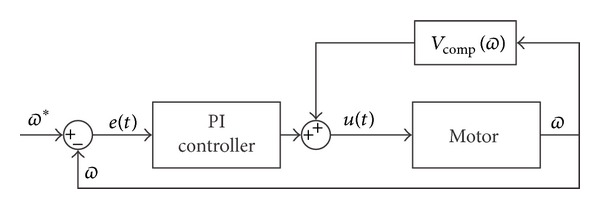
PI control strategy for a DC motor.

The control diagram for each motor can be seen in Figure 14. 

### 3.3. Hierarchical Control Integration

**Figure 15:**
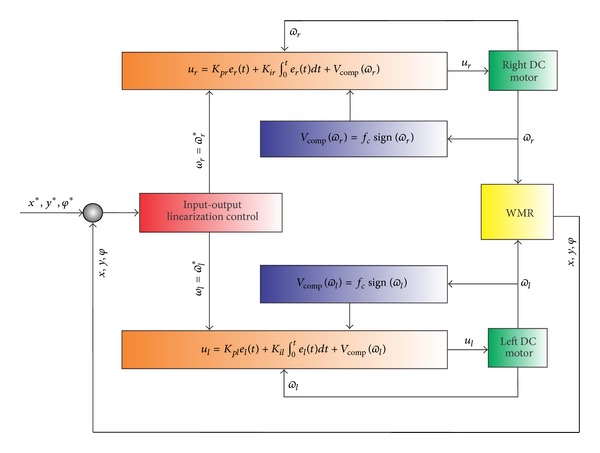
WMR hierarchical control block diagram.

 The control law described before has considered only the kinematic structure of the WMR, Section 3.1, and the actuator system in Section 3.2, and both systems are basically the WMR. In order to present clearly the integration of the hierarchical control, Figure 15 shows a block diagram for this purpose. 

Regarding the DC motor control, the nominal output ϖ* used in (38) is determined for the angular path required *ϖ*_*r*_ or *ϖ*_*l*_. Since the WMR has two equal and independent motors (left and right), the dynamic models are given by

(39) dϖrdt=−10.20ϖr+5.51ur, yr=ϖr,dϖldt=−10.20ϖl+5.99ul, yl=ϖl,

where *ϖ*_*r*_ and *ϖ*_*l*_ are the right and left angular velocity of the motors.

Thus, two controls, *u*_*r*_ and *u*_*l*_, are required; according to Section 3.2, they are given by (38), so that

(40) ur=Kprer(t)+Kir∫0ter(t)dt+Vcomp(ϖr),ul=Kplel(t)+Kil∫0tel(t)dt+Vcomp(ϖl),

with *V*_comp_(*ϖ*_*r*_) and *V*_comp_(*ϖ*_*l*_), defined by

(41) Vcomp(ϖr)=fcsign⁡(ϖr),Vcomp(ϖl)=fcsign⁡(ϖl),

where *u*_*r*_ and *u*_*l*_ are the control voltages for the right and left motors, respectively, *K*_*pr*_ and *K*_*ir*_, *K*_*pl*_ and *K*_*il*_, are the constant gains (proportional and integral) associated to each motor. Finally, *e*_*r*_(*t*) and *e*_*l*_(*t*) represent the motor tracking errors defined by

(42)   er(t)=ϖr∗−ϖr,  el(t)=ϖl∗−ϖl,(ϖr∗,ϖl∗)=(ωr,ωl).

It means that the desired angular velocity profiles for the motors, (*ϖ*_r_*,  *ϖ*_l_*), are determined by the (*ω*_*r*_, *ω*_*l*_). These angular velocity trajectories are obtained from (15) and (16).

## 4. Trajectory Generation via the Method of Interpolation of Cubic Splines

 As can be seen from (15) and (16), the synthesis of these formulations requires the knowledge of the desired trajectory, i.e., (x*, y*, φ*). This section presents the path generation problem solved via the cubic splines interpolation approach, in order to extend the possible trajectories to be followed (compared to the parametric approach widely reported in the literature).

### 4.1. Cubic Splines

Polynomial interpolation is based on a substitution of either a mathematical function or a lookup table for a polynomial. The bigger the number of points, the higher the degree of the polynomial, and consequently the greater the error between the desired trajectory and the interpolated curve due to oscillations. An alternative method is to employ several polynomials of lower degree, in subintervals along the whole trajectory. This method is the basis of the spline interpolation approach [21–23]. The method divides the path to be tracked into subintervals (given by the lookup table points) and a cubic interpolation joins such subintervals. This method is known as polynomial segmentary approximation as

(43) $$\begin{array}{c} S_{j}\left(x\right)=a_{j}+b_{j}\left(x-x_{j}\right)+c_{j}{\left(x-x_{j}\right)}^{2}+d_{j}{\left(x-x_{j}\right)}^{3}. \end{array}$$

For each *j* = 0,1,…, *n* − 1 and according to [21], the equation used for cubic spline interpolation is given by *Ax* = *b*, where

(44) A=[100…0h02(h0+h1)h1…00h1        2(h1+h2)h20……………0…hn−2        2(hn−2+hn−1)hn−10…001],b=[03h1(a2−a1)−3h0(a1−a0)⋮3hn−1(an−an−1)−3hn−2(an−1−an−2)0],x=[c0c1c2⋯cn]T,

where *A* is an (*n* − 1) by (*n* − 1) diagonal matrix, *b* is a constant vector, and *x* is an unknown vector. A numerical method is used to find *c*_0_, *c*_1_,…, *c*_*n*_, and based on this method, the coefficients in (43), *a*_*j*_, *b*_*j*_, *c*_*j*_, and *d*_*j*_, are calculated in order to obtain a cubic polynomials for each segment.

### 4.2. Interpolation Algorithm

Based on the previous approach, a path planning algorithm is presented in this section. This method generates the Cartesian coordinates of the points along the path to be followed: these points are given by (x*, y*, φ*), using (15) and (16) in order to allow (*x*, *y*, *φ*)→(x*, y*, φ*).

**Algorithm**

In order to build the cubic interpolation function (43), according to [21], there must be defined the number of points *n* which satisfy the following condition: *x*_0_ < *x*_1_ < ⋯<*x*_*n*_ with $\ddot{S}(x_{0})=\ddot{S}(x_{n})=0$. The following steps are suggested to generate (x*,  y*,  φ*).

 (1) Vector coordinate points are introduced, so that, *x*_0_, *x*_1_,…, *x*_*n*_ and *y*_0_, *y*_1_,…, *y*_*n*_.
 (2) Assign *a*_0_ = *f*(*x*_0_), *a*_1_ = *f*(*x*_1_),…, *a*_*n*_ = *f*(*x*_*n*_).
 (3) For *i* = 0 until *n* − 1, set *h*_*i*_ = *x*_*i*+1_ − *x*_*i*_.
 (4) For *i* = 1 until *n* − 1, set *α*_*i*_ = (3/*h*_*i*_)(*a*_*i*+1_ − *a*_*i*_) − (3/*h*_*i*−1_)(*a*_*i*_ − *a*_*i*−1_).
 (5) Put *l*_0_ = 1,  *μ*_0_ = 0,  *z*_0_ = 0.
 (6) For *i* = 1 until *n* − 1, set
  (45) li=2(xi+1−xi−1)−hi−1μi−1,μi=hili,zi=(αi−hi−1zi−1)li.
 (7) Set *l*_*n*_ = 1,  *z*_*n*_ = 0,  *c*_*n*_ = 0.
 (8) For *j* = *n* − 1 until 0, set
  (46) cj=zj−μjcj+1,bj=(aj+1−aj)hj−hj(cj+1+2cj)3,dj=(cj+1−cj)3hj.
 (9) If *x* ≤ *x*_0_, then *j* = 1; otherwise, *x* ≥ *x*_0_ and *x* ≤ *x*_1_ and then *j* = 2; otherwise, *x* ≥ *x*_*n*−1_ and *x* ≤ *x*_*n*_ and then *j* = *n* − 1.
 (10) If *x* ≤ *x*_*n*_, then
  (47) $$\begin{array}{c} y_{j}=a_{j}+b_{j}\left(x-x_{j}\right)+c_{j}{\left(x-x_{j}\right)}^{2}+d_{j}{\left(x-x_{j}\right)}^{3}. \end{array}$$
 (11) Based on the equation obtained in step 10, the following expression is obtained:
  (48) $$\begin{array}{c} {\dot{y}}_{j}=b_{j}+2c_{j}\left(x-x_{j}\right)+3d_{j}{\left(x-x_{j}\right)}^{2}. \end{array}$$
 (12) φ* is obtained as $\varphi^{*}=\arctan(\dot{y})$.
 (13) Based on this, considering the points (1), (10), and (12), the coordinates obtained are denoted by (x*, y*, φ*).

This algorithm is encoded in MATLAB in order to implement experiments using the WMR.

## 5. Experimental Results

 This section presents the closed-loop simulation as well as experimental results in order to validate the behavior of the hierarchical approach presented in Section 3, for both parametric and generated (based on given points) curves. The simulations are implemented in MATLAB/Simulink, and the real-time experiments are based on the board DS1104 using the same software.

 According to the WMR dimensions, *r* and *l* have the following values:

(49) $$\begin{array}{c} r=0.075\;\text{m},\;\;l=0.22\;\text{m}. \end{array}$$

Regarding the motors, whose characterization was presented in Section 3.2, the right motor has the following parameters:

(50) $$\begin{array}{c} K_{r}=0.54,\;\;\tau_{r}=0.10. \end{array}$$

The left motor has the parameters

(51) $$\begin{array}{c} K_{l}=0.59,\;\;\tau_{l}=0.10. \end{array}$$

The controller gains (15) and (16) and the gains of the controllers (40), were selected like:

(52) $$\begin{array}{c} \alpha_{x}=\alpha_{\varphi }=2,\;\;K_{pr}=K_{pl}=2,\;\;K_{ir}=K_{il}=50. \end{array}$$

### 5.1. Experimental Results Using the Parametric Curve Approach

General results are used when the desired path is a parabola, so that

(53) $$\begin{array}{c} y^{*}\left(t\right)={x^{*}}^{2}\left(t\right). \end{array}$$

The parametrization is chosen as follows:

(54) $$\begin{array}{c} x^{*}\left(t\right)=A\sin\left(\frac{2\pi }{P}t\right). \end{array}$$

It is found that φ*(*t*) is determined by the following equation:

(55) $$\begin{array}{c} \varphi^{*}\left(t\right)=\arctan\left(2x^{*}\left(t\right)\right). \end{array}$$

This parametrization allows the displacement of the WMR from the origin to the point (*x*, *y*) = (*A*, *A* ^2^), later to (*x*, *y*) = (−*A*, *A* ^2^) passing through the origin and finally returning to the origin in *P* seconds. The parameter values *A* and *P* are associated to (54), and they are chosen to be

(56) $$\begin{array}{c} A=1\;\text{m},\;\;P=15\;\text{s}. \end{array}$$

**Figure 16:**
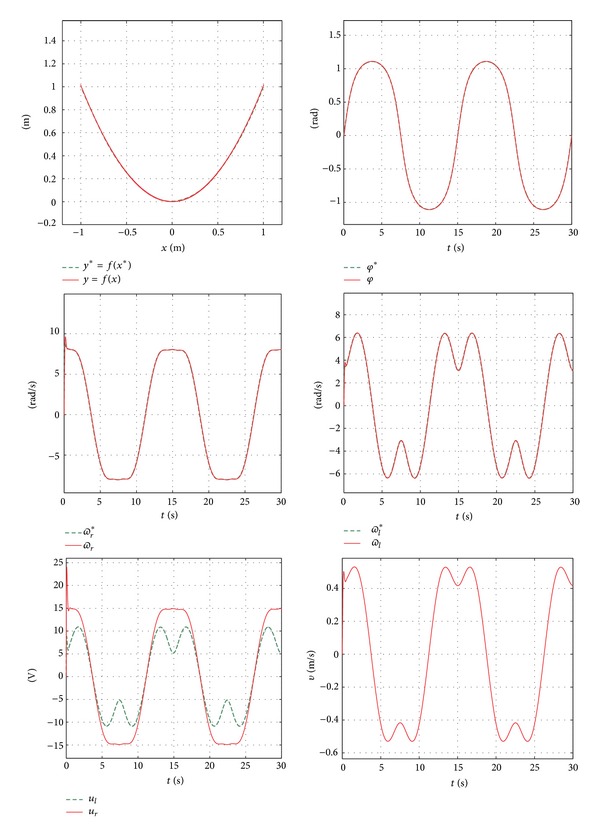
Simulation results.

**Figure 17:**
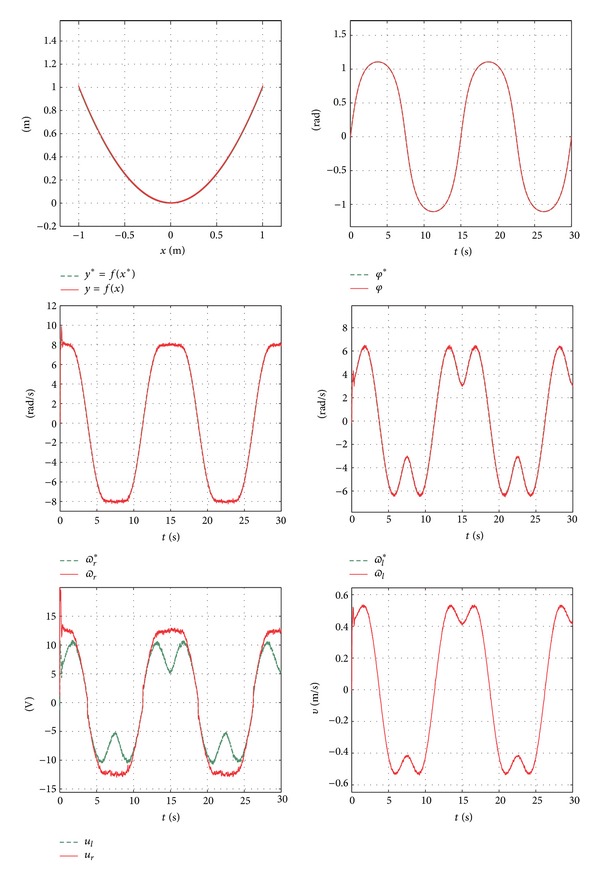
Real-time experimental results.

In order to simulate the system, an initial condition chosen for (*x*, *y*, *φ*) is (0,0, 0), assuming *t* = 30 s, where *t* is the total simulation time. The simulation results are presented in Figure 16. Similarly, the real-time experiments require the same conditions used in the simulation. The data was obtained using ControlDesk and MATLAB/Simulink along with the DS1104 board. These results are presented in Figure 17, and Video S4 in Supplementary Material. 

### 5.2. Experimental Results Using Generated Curves Given by Points

**Figure 18:**
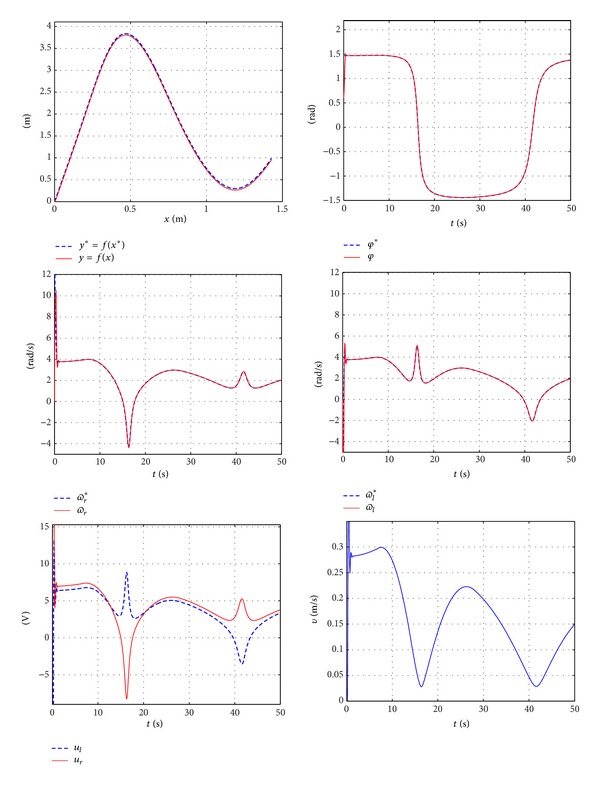
Simulation results for a curve obtained from given points.

**Figure 19:**
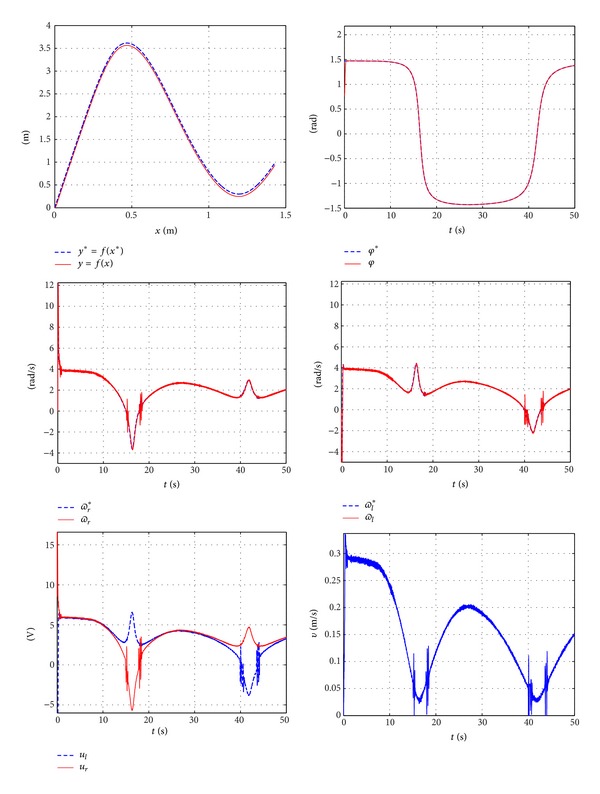
Experimental results for curve obtained by points.

This section presents both the experimental and the simulation results obtained in Section 3 where the curve generated is obtained from coordinate points. The chosen points are given by Table 1. Both the control system and the parameters used in the previous subsection are employed, (49)–(52), providing the simulation results shown in Figure 18. The experimental results are shown in Figure 19. 

### 5.3. Discussion of the Experimental Results

Similar results are obtained in both the simulation and the experimental plots. The velocity is estimated based on the position measured directly from the encoders attached to the motor shafts. Both *ϖ*_*r*_ and *ϖ*_*l*_, follow the desired path angular velocity according to the kinematic structure *ϖ*_r_* and *ϖ*_l_* respectively. Consequently, trajectory tracking task is performed successfully, (i.e., (*x*, *y*, *φ*) → (x*, y*, φ*)). In addition, it is shown that the control voltages *u*_*r*_ and *u*_*l*_ are within the interval (−24 V, +24 V), which is convenient since the nominal motor voltages are in the range of ±24 V. Also, from the results, we find that the maximum linear value *v* is equal to 0.52 m/s. Based on all the previous results, the efficiency of the hierarchical control design has excellent performance.

## 6. Conclusions

This work shows the design and construction of a wheeled mobile robot with differential configuration architecture. In addition, a planning trajectory has been proposed and implemented with a control law that experimentally showed successful results, for two approaches: smooth parametric trajectories and interpolated trajectories determined by given points.

More specifically, this control is hierarchically distributed in two controllers: the first controller is meant to use the kinematic model of the WMR, which is based on a linearization of the input-output plant (*ω*_*r*_, *ω*_*l*_)-(*x*, *φ*), that generates a remaining dynamics for *y* that is stable. The second controller is a PI strategy that determines the output control signals in order to track the desired velocities *ω*_*r*_ and *ω*_*l*_ such that *x* → x*, *y* → y*, and *φ* → φ*, with the initial conditions *x* = 0, *y* = 0, and *φ* = 0. The main difference between this hierarchical control and the nonlinear techniques is that these last ones have to commutate between two control laws in order to keep the system stable. In this approach, there is a drawback to the use of parametric trajectories, which happens when *φ* = *kπ*/2 for *k* = ±1, ±3, ±5,…. This is a significant constraint when the trajectory to be followed is a parametric curve, since the controller, in some conditions, is undetermined. For this reason, the validation of the results was carried out not only with the use of the parametric curves approach, but also with an interpolation method based on cubic splines, which enables following a wider range of trajectories than is possible with parametric curves.

The second controller is designed for the model of the motors given by (39), and this control law guarantees the trajectory tracking given for the desired trajectory established for the first controller. The so-called * hierarchical approach* was introduced in [10] and used in [16] and was graphically presented in Figure 15.

Likewise, this research found that a control based on an input-output plant linearization approach for (*ω*_*r*_, *ω*_*l*_)-(*y*, *φ*) is always possible in such a way that *φ* ≠ *kπ* for *k* = 0, ±1, ±2,…, since the matrix at these points becomes singular.

The synergetic combination of the theoretical results, implementation techniques, and construction description is the main contribution of this research. This approach has also an important impact for mathematicians willing to explore the application field as well as practical researchers who might like to know more about the theoretical results described in a real and effective prototype.

Finally, as possible direction for future research, this approach can be extended to the problem of real-time obstacle avoidance. Similarly, another possible direction for future research is the modification of the second controller (related to the actuators), where the armature voltage and current can be the feedback variables for velocity control instead of the encoders; this then will become a speed sensorless approach.

## Supplementary Material

Supplementary Video 1 and Supplementary Video 2: Materials used for the prototype construction. These videos show how some parts of the WMR prototype were built. Different machine tools were employed for the construction.
Supplementary Video 3: This video shows different real views of the complete WMR prototype. These included the mechanical and electronic stages.
Supplementary Video 4: This video shows the real-time experiments associated with the case when the desired trajectory is defined by a parabola equation. The experiments results were obtained using ControlDesk and MATLAB/Simulink along with the DS1104 board from dSPACE.

## Figures and Tables

**Table 1:** Points used in order to generate the desired trajectory.

x*	y*
*x*_0_ = 0.0 m	*y*_0_ = 0.0 m
*x*_1_ = 0.2 m	*y*_1_ = 0.2 m
*x*_2_ = 0.4 m	*y*_2_ = 3.5 m
*x*_3_ = 0.8 m	*y*_3_ = 2.0 m
*x*_4_ = 1.2 m	*y*_4_ = 0.3 m
*x*_5_ = 1.4 m	*y*_5_ = 1.0 m
